# A novel *TAB2* mutation detected in a putative case of frontometaphyseal dysplasia

**DOI:** 10.1038/s41439-021-00166-6

**Published:** 2021-10-29

**Authors:** Asuka Hori, Ohsuke Migita, Rika Kawaguchi-Kawata, Yoko Narumi-Kishimoto, Fumio Takada, Kenichiro Hata

**Affiliations:** 1 Department of Medical Genetics and Genomics, the Kitasato University Graduate School of Medical Sciences, Kanagawa, Japan; 2 Department of Genetics and Genomics, the Kitasato University Hospital, Kanagawa, Japan; 3 Department of Maternal-Fetal Biology, Research Institute, National Center for Child Health and Development, Tokyo, Japan; 4 Faculty of Medicine, University of Tsukuba, Ibaraki, Japan

**Keywords:** Disease genetics, Genetic testing

## Abstract

Frontometaphyseal dysplasia (FMD) type 2 is an autosomal dominant disorder characterized by skeletal abnormalities and caused by *MAP3K7* mutation. We identified a novel missense mutation in *TAB2* associated with FMD in a child with multiple congenital malformations. This case was diagnosed as FMD due to joint contractures and bone deformities. This is the third report of FMD caused by a *TAB2* mutation located in the TAK1-binding region.

Frontometaphyseal dysplasia (FMD) is a progressive osteosclerotic disease affecting the long bones and skull (OMIM #305620, OMIM #617137). FMD is either an X-linked or an autosomal dominant disorder; *FLNA*^1^ on the X chromosome and *MAP3K7*^2^ on chromosome 6, respectively, are responsible for the disease. Recently, a mutation in the TGF-β-activated kinase 1 and MAP3K7-binding protein 2 (*TAB2)* gene was reported in a case of FMD. TAB2 binds TGF-β-activated kinase 1 (TAK1), the product of *MAP3K7*. Overexpression of TAK1, which is involved in multiple signaling pathways, is speculated to be associated with FMD type 2^2^. Although the clinical manifestations of *FLNA* and *MAP3K7* mutations are similar, cases with *MAP3K7* mutation are more likely to display keloid formation, characteristic facial features, hearing impairment, scoliosis, and cervical fusion^3^. In addition, despite no reports of intellectual disability in patients carrying *FLNA* mutation, there are several reports of intellectual disability in cases with *MAP3K7* mutation. Recently, a *TAB2* mutation was reported in two subjects with clinical symptoms of FMD^2,3^. In this study, we identified a novel missense mutation in *TAB2* in a case clinically consistent with FMD. Here, we summarize the symptoms of FMD caused by *TAB2* mutation.

The patient was a girl (Fig. 1a, III-2) born as the first child of unrelated Japanese parents (Fig. 1a, II-2, and II-5). The mother had a history of one miscarriage. The patient was born by cesarean section due to nonreassuring fetal status at 40 weeks and 6 days after a diagnosis of amniotic fluid deficiency. She weighed 2762 g; she was 50.0 cm long and 32.0 cm in the cephalic position. At birth, she had a high-pitched cry, flabby eyelids, micrognathia, an enlarged fontanel, and abnormal alignment of the bilateral second toes. No abnormalities on newborn hearing screening or on head MRI performed at the age of 13 days were noted. At 2 months of age, she was found to have restricted hip opening, patent ductus arteriosus (PDA), and atrial septal defect (ASD). Chromosome test results were 46,XX. She started sitting at the age of 8 months and turned at the age of 9 months. At the age of 11 months, she was unable to rotate both elbows but had limited extension and limited arm elevation due to winged scapula. By then, she was found to have bilateral clubfeet and a thick femoral neck on X-ray images. At 1 year and 2 months of age, she started wearing glasses due to refractive errors in both eyes, and ptosis and external strabismus were also noted. At 1 year and 3 months of age, she underwent surgical treatment for ASD and PDA. At 1 year and 4 months of age, she could walk independently. At the age of 2 years, she uttered a sentence consisting of two words. She had an abnormal ECG and keloid formation at a postoperative wound site. She also exhibited features of brachycephaly, short neck, distal interphalangeal joint contractures of all fingers and toes, mild scoliosis, coarse face, prominent supraorbital ridge, down slanting palpebral fissures, thick eyebrows, hypertelorism, flat and broad nasal root, short small nose, long philtrum, and low-set and posteriorly rotated ears.

**Fig. 1:**
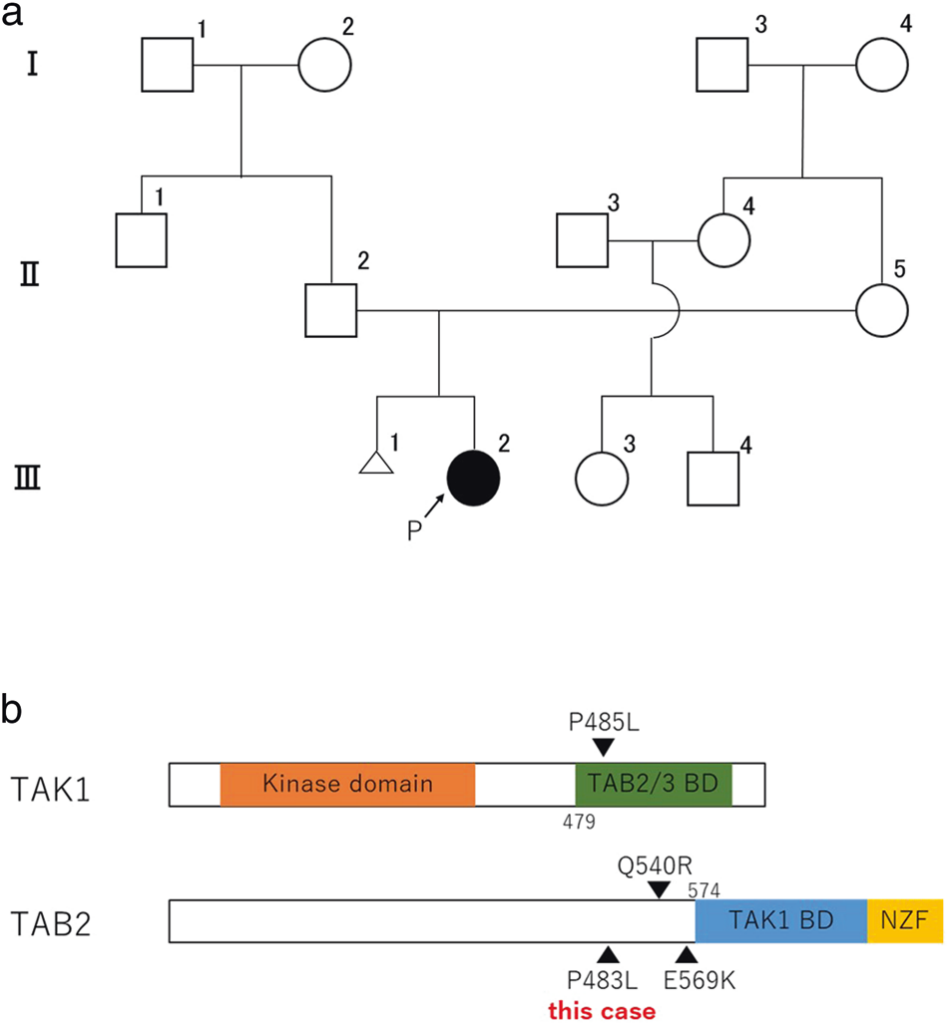
Information on the patient and the missense TAB2 mutation. **a** A family pedigree of the patient with FMD. **b** Schematic representation of the domain structures of human TAK1 and TAB2. TAB2/3 BD: TAB2/TAB3-binding domain, TAK1 BD: TAK1-binding domain, NZF: Np14 zinc finger.

Based on the family’s intention to search for the cause of the congenital anomaly, molecular genetic analysis of the affected child and her parents was performed. The study was approved by the ethics committees of the National Center for Child Health and Development and Kitasato University Hospital, and written consent was obtained from the parents. DNA was extracted from peripheral blood of II-2, II-5, and III-2 (Fig. 1a), whole-exome sequencing (WES) was performed, and whole-exome libraries were prepared using Agilent SureSelect v6 Capture Kit (Agilent Technologies, Santa Clara, CA, USA) according to the manufacturer’s protocol. The libraries were sequenced using an Illumina HiSeq2500 in paired-end mode at 101 bp. Sequences were examined according to laboratory procedures^4^. Variant frequencies were obtained from the 1000 Genomes Project database (http://www.internationalgenome.org), Human Genome Variation Database (HGVD; http://www.hgvd.genome.med.kyoto-u.ac.jp), and Japanese Multi Omics Reference Panel (jMorp; https://jmorp.megabank. tohoku.ac.jp).

WES detected heterozygosity of NM_001292034.3:c.1448 C > T :p.Pro483Leu in *TAB2* only in III-2; this mutation was not found in her parents (Fig. 1a, II-2, and II-5). Similar results were obtained by Sanger sequencing. This is a novel mutation not registered as a pathological variant in dbSNP (https://www.ncbi.nlm.nih.gov/snp/), ClinVar (https://www.ncbi.nlm.nih.gov/clinvar/), or Human Genome Mutation Database (HGMD; http://www.hgmd.cf.ac.uk/). This domain is highly preserved, and the mutation is predicted to be damaging by the protein function prediction software SIFT and PolyPhen-2. It is not described in the general population-sequencing information such as gnomAD (https://gnomad.broadinstitute.org/), 1000 Genomes Project database, HGVD, or jMorp. The pathogenicity of the variant was assessed according to ACMG guidelines^5^, with PS2 (de novo), PM2 (evidence in population database), PP3 (computational evidence), and PP4 (phenotype specific for gene) relevant evidence. Therefore, we classified the variant as likely pathogenic.

Nonsyndromic and missense variants of TAB2 have a putative role in congenital heart defects^6^ and are located outside the TAK1-binding domain. Missense TAB2 variants have also been reported in two patients with clinical symptoms of FMD^3^. Although FMD is known to be caused by *FLNA* and *MAP3K7* heterozygous mutations, *TAB2* has not yet been a confirmed causative gene. Nevertheless, TAK1 and TAB2 have junctional domains, and P485L, frequently reported in FMD with MAP3K7 variants, is located in the TAB2-binding domain^2^. Two missense variants, Q540R and E569K, were recently reported in cases of FMD with *TAB2* mutations (Fig. 1b); both are located adjacent to the TAK1-binding domain in a region known as the coiled-coil region^7^. The present case involved the missense mutation c.1448 C > T (P483L), which is located slightly toward the N-terminus from these variants.

Based on the function of the gene product, FMD with *TAB2* mutation may present a clinical picture similar to that caused by *MAP3K7* mutation^3^. A summary of the clinical presentation of our subject is given in Table 1, along with previously reported cases of FMD. Our case differs from the other two cases in that mild keloid formation and congenital heart disease (ASD and PDA) were present. However, as keloid formation is an FMD type 2 characteristic^2^ and *TAB2* is associated with congenital heart disease, it is possible that the symptoms in our subject were due to the *TAB2* mutation. In FMD type 2 patients, intellectual disability is reported in some cases; hearing impairment has also been reported in a high percentage of cases. In the present case and the previously reported cases of *TAB2* mutation, no obvious developmental delay was noted, and no hearing impairment was confirmed. This may be related to functional differences in the *TAB2* gene, though further cases are needed for verification.

**Table 1:** Summary of clinical symptoms of reported frontometaphyseal dysplasia.

	FLNA	MAP3K7	TAB2	This case
Prominent supraorbital ridges	+	+	+	+
Hypertelorism	+	+	+	+
Down slanting palpebral fissure	±	+	+	+
Broad bridge of command	+	+	+	+
Micrognathia	±	+	+	+
Deafness	±	+	±	−
Cardiac anomaly	±	±	−	+
Finger/wrist contracture	+	+	+	+
Elbow contracture	±	+	+	+
Broad finger	±	+	±	+
Scoliosis	±	+	+	+
Cervical spine fusion	−	±	±	−
Long fingers	±	+	+	−
Keloid	NA	±	−	+
Impaired intelligence	−	±	−	−

+: Present, –: absent, ±: both presence and absence were reported, *NA* not available.

A novel variant of *TAB2* detected in a putative case of frontometaphyseal dysplasia.

In conclusion, we identified a novel missense mutation in *TAB2* that may cause FMD. TAK1, which is known to be causative in FMD type 2, and TAB2 bind together, and dysfunction of the TAK1–TAB2 complex is involved in TAK1-related diseases^8^. The present FMD case carried a *TAB2* mutation, as reported in two previous cases, strongly supporting the possibility that *TAB2* mutation may cause FMD. The naming of ‘FMD3’ has been proposed, as *TAB2* mutation represents a third form of FMD^2^. However, the difference in phenotype between FMD2 caused by *MAP3K7* mutation and FMD caused by *TAB2* mutation has not yet been clarified. Symptoms of this disease are progressive, and clinical diversity is expected in FMD due to TAB2 variants. There are no phenotypic data for patients over 40 years of age^3^, and it is important to accumulate cases and record natural history based on genotyping of FMD type 2 and FMD due to *TAB2* mutation. In addition, the fact that variants in multiple genes cause similar symptoms suggests the need for a biochemical understanding of gene interactions and pathogenesis. Investigating factors that affect these interactions is also needed for the development of drugs that can cure or alleviate symptoms.

## Data Availability

The relevant data from this Data Report are hosted at the Human Genome Variation Database at 10.6084/m9.figshare.hgv.3036.
